# CircHPCAL1 promotes the progression of pancreatic cancer via the regulation of STEAP2

**DOI:** 10.1002/ctm2.70501

**Published:** 2025-10-09

**Authors:** Xiaomeng He, Yang Di, Lixiang Sun, Wenchuan Wu, Zehuan Li, Qiuyue Li, Shanshan Liu, Mengting Luo, Xin Zhang, Li Xu, Xiaoyan Zhang, Jianqing Xu, Christopher Corpe, Jin Wang

**Affiliations:** ^1^ Central Laboratory, Zhongshan Hospital (Xiamen) Fudan University Xiamen China; ^2^ Shanghai Public Health Clinical Centre Fudan University Shanghai China; ^3^ Department of Pancreatic Surgery, Pancreatic Disease Institute, Huashan Hospital, Shanghai Medical College Fudan University Shanghai China; ^4^ Xiamen Key Laboratory of Biotherapy Xiamen China; ^5^ Department of General Surgery, Zhongshan Hospital Fudan University Shanghai China; ^6^ Clinical Research Centre for Precision Medicine of Abdominal Tumour of Fujian Province Xiamen China; ^7^ Department of Nutritional Science King's College London London UK

1

Dear Editor,

Pancreatic cancer (PaCa) has emerged as the fourth leading cause of cancer‐related mortality, accounting for approximately 8% of all cancer deaths globally.^1^ This high mortality rate is attributed primarily to the late diagnosis of PaCa due to the absence of discernible early symptoms.^2^ Circular RNAs (circRNAs) play pivotal roles in various cellular biological processes and have emerged as promising diagnostic biomarkers and therapeutic targets.^3^, ^4^, ^5^, ^6^ The crucial roles of circRNAs in processes such as cell proliferation, migration and invasion have been identified in many cancer cells.^7^, ^8^, ^9^ Accumulating evidence has revealed that circRNAs are involved in PaCa progression and have shown promise as diagnostic biomarkers for PaCa.^10^

In this study, we revealed that a novel circRNA (circHPCAL1) identified in the plasma of patients with PaCa using high‐throughput circRNA sequencing was also upregulated in pancreatic tumour tissues compared with that in adjacent normal tissues from 78 PaCa patients (Figure 1A). Similarly, a panel of PaCa cell lines (BxPC‐3, CFPAC‐1, AsPC‐1, PANC‐1 and KP‐3) presented higher expression levels of circHPCAL1 than normal HPNE cell did (Figure 1B). Because circHPCAL1 originates from exon 1 and a portion of the 5′ untranslated region of the HPCAL1 gene, we designed specific divergent and convergent primers for PCR amplification (Figure 1C) and found that circRNAs could be amplified by both divergent and convergent primers, whereas genomic DNA could be amplified via only convergent primers. RNA fluorescence in situ hybridisation analysis revealed that circHPCAL1 is predominantly localised in the cytoplasm of BxPC‐3 and PANC‐1 cells (Figure 1D). Upregulation of circHPCAL1 significantly increased the proliferation and viability of BxPC‐3 and PANC‐1 cells, whereas si‐circHPCAL1 inhibited PaCa cell growth (Figure 1E,F). The colony‐forming ability of BxPC‐3 and PANC‐1 cells was significantly augmented by circHPCAL1 upregulation and impaired by the circHPCAL1 inhibitor (Figure 1I–K). The migration and invasion abilities of BxPC‐3 and PANC‐1 cells were also analysed via wound healing (Figure S1A–C) and Transwell (Figure 1L,M) assays. We also treated BxPC‐3 and PANC‐1 cells with gemcitabine and found that overexpression of circHPCAL1 attenuated the sensitivity of BxPC‐3 (IC_50_ = 2.093 µM) and PANC‐1 (IC_50_ = 15.610 µM) cells to gemcitabine, whereas si‐circHPCAL1 increased their sensitivity (BxPC‐3: IC_50 _= 0.694 µM; PANC‐1: IC_50 _= 3.986 µM) (Figure 1G,H).

**FIGURE 1:**
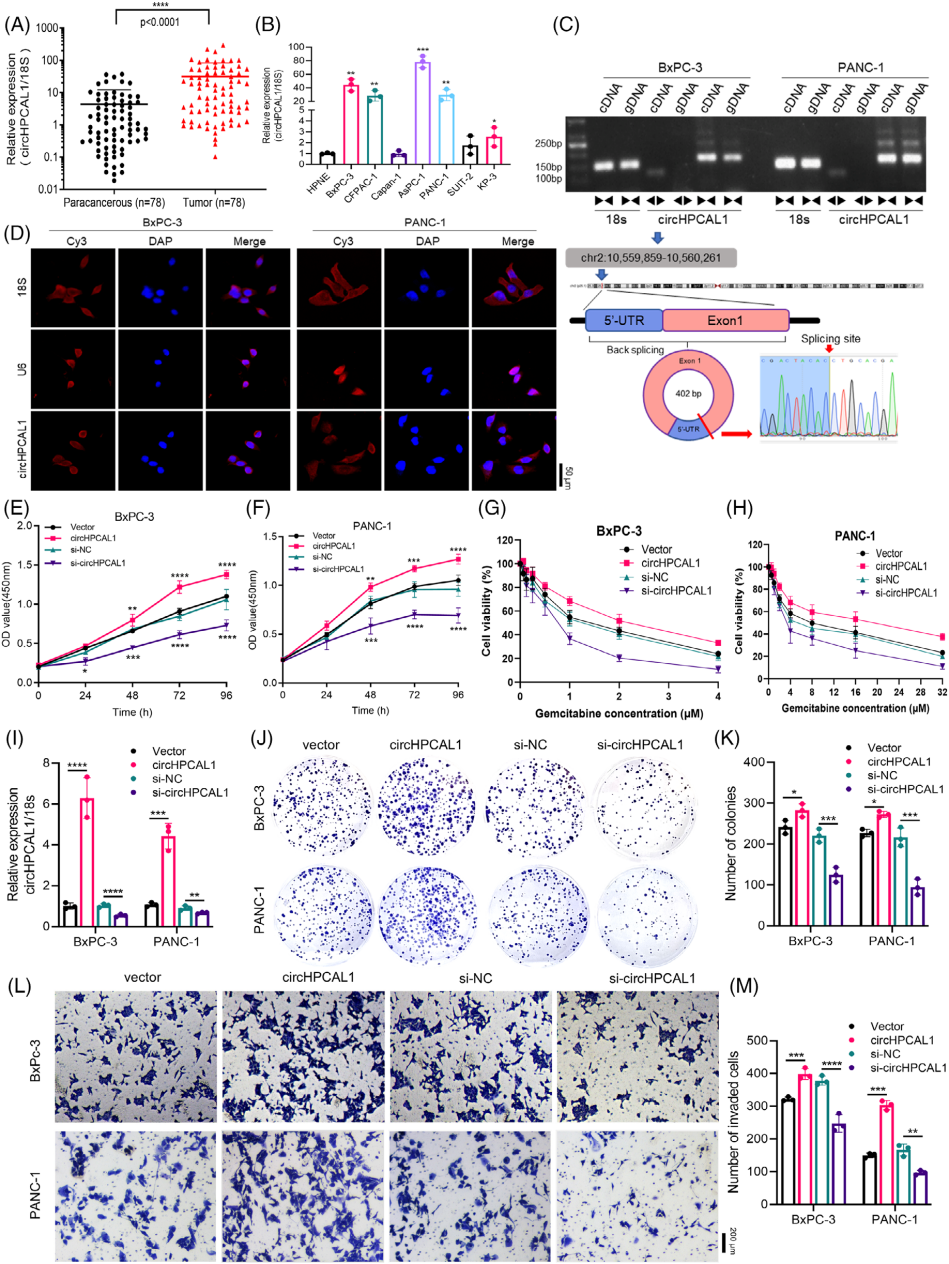
CircHPCAL1 expression is upregulated in pancreatic tumour tissue and cells and promotes the malignant behaviour of pancreatic cancer (PaCa) cells. (A, B) The expression levels of circHPCAL1 were analysed in pancreatic tumour tissues (A) and cells (B) by quantitative real‐time polymerase chain reaction (qRT‐PCR). (C) Schematic diagram of the circular structure of circHPCAL1 and confirmation of its circular structure via PCR and Sanger sequencing. (D) RNA‐FISH showed the subcellular localisation of circHPCAL1 in PaCa cells. (E, F) The CCK8 assays revealed that circHPCAL1 promotes the proliferation of PaCa cells. (G, H) The cell viability assays of the overexpression or knockdown of circHPCAL1 in PaCa cells or treatment with gemcitabine. (I) The expressed levels of circHPCAL1 in PaCa cells with overexpression or siRNAs targeting circHPCAL1. (J–M) The colony formation (J, K) and Transwell assays (L, M) were used to evaluate the effects of circHPCAL1 in PaCa cells. * *p* < 0.05, **p < 0.01, ***p < 0.001 and ****p < 0.0001.

To identify potential target miRNAs of circHPCAL1, 11 overlapping candidate miRNAs were selected via starBase, circBank, and circRNA interactome analyses (Figure 2A). Overexpression of circHPCAL1 led to decreased expression of miR‐671‐5p, miR‐338‐3p, and miR‐7‐5p in BxPC‐3 and PANC‐1 cells, whereas knockdown of circHPCAL1 increased their expression (Figure 2B, 2C). We further found that only miR‐671‐5p decreased luciferase activity specifically via the circHPCAL1 binding site but not its mutated form, confirming a direct interaction between circHPCAL1 and miR‐671‐5p (Figure 2D, 2E, S2A, and S2B). miR‐671‐5p was also significantly downregulated in both PaCa cell lines (Figure 2F, S2C, S2D) and tissue samples according to quantitative real‐time polymerase chain reaction (qRT‐PCR) analyses (Figure 2G). K‐M analysis revealed that lower miR‐671‐5p expression was associated with shorter survival in PaCa patients (Figure 2H). To investigate the interaction between circHPCAL1, miR‐671‐5p and AGO2, RNA immunoprecipitation (RIP) assays were conducted in BxPC‐3 and PANC‐1 cells with anti‐AGO2 antibodies. The results revealed that circHPCAL1 and miR‐671‐5p were significantly enriched in cells overexpressing miR‐671‐5p, with IgG serving as a negative control (Figure 2I,J). In addition, growth curve (Figure 2K,L) and colony formation (Figure 2M,N) assays demonstrated that upregulation of miR‐671‐5p significantly inhibited the proliferation and viability of these cells, whereas downregulation of miR‐671‐5p promoted cell growth. Transwell (Figure 2O,P) and wound healing (Figure S2E–G) assays revealed that the overexpression of miR‐671‐5p inhibited PaCa cell invasion and migration. Our findings suggest that miR‐671‐5p, as the target miRNA of circHPCAL1, plays a tumour‐suppressive role in PaCa. RNA sequencing was next used to explore the potential molecular mechanisms underlying the role of circHPCAL1 in PaCa progression. Based on the criteria of |logFC| > 1 and *p* < 0.05, we identified a total of 118 DEGs (Table S2), with 27 genes upregulated and 91 genes downregulated (Figure 3A). Combined with the analyses of the miRDB database, only eight genes were found to be potential targets of miR‐671‐5p (Figure 3B). Finally, we demonstrated that overexpression of circHPCAL1 led to upregulation of STEAP2 in both BxPC‐3 and PANC‐1 cells (Figure 3C,D), and the cotransfection of HEK293T cells with a luciferase reporter containing the binding site of the STEAP2 3′UTR and miR‐671‐5p resulted in a significant reduction in luciferase intensity, whereas a reporter with a mutated binding site was unaffected (Figure 3E,F). circHPCAL1 overexpression also increased STEAP2 protein levels in BxPC‐3 and PANC‐1 cells, whereas si‐circHPCAL1 decreased STEAP2 protein levels (Figure 3G). Conversely, the overexpression of miR‐671‐5p reduced STEAP2 protein expression (Figure 3H). Immunofluorescence assay revealed the colocalization of circHPCAL1 and STEAP2 protein in the cytoplasm of PaCa tumour tissue (Figure 3I). In addition, we found that the protein levels of STEAP2 were high or moderate in the cytoplasm and membrane of PaCa tumours via the Human Protein Atlas (Figure 3J). High STEAP2 expression levels in PaCa cell lines (Figure 3K) and tumour tissue samples were investigated via qRT‒PCR analyses (Figure 3L) and GEPIA2 (Figure 3 M). Our results indicated that STEAP2 could be a target of miR‐671‐5p and was upregulated in PaCa by circHPCAL1. Furthermore, growth curve (Figure 3N–P) and colony formation (Figure S3A,B) assays demonstrated that the upregulation of STEAP2 significantly increased PaCa cell proliferation and viability. Transwell (Figure 3Q,R) and wound healing (Figure S3C–E) assays revealed that STEAP2 overexpression significantly promoted PaCa cell invasion and migration, whereas STEAP2 knockdown inhibited these phenomena. Through gene function enrichment analysis, we found that circHPCAL1 was related to PI3K/Akt (NES (standardised enrichment score) = 1.557, *p *= 0.005) and the mTOR pathway (NES = 1.522, *p* = 0.007) (Figure 3S,T). Overexpression of STEAP2 in BxPC‐3 and PANC‐1 cells led to the marked upregulation of phosphorylated PI3K (p‐PI3K), phosphorylated AKT (p‐AKT), and phosphorylated mTOR (p‐mTOR) proteins. We found that a significant increase in the protein expression of vimentin and SNAIL1 and a notable decrease in E‐cadherin protein expression in PaCa cells (Figure 3U). Furthermore, circHPCAL1 overexpression in PaCa cells significantly upregulated STEAP2 protein expression, which was accompanied by increased levels of p‐PI3K, p‐AKT, p‐mTOR, vimentin, and SNAIL1 proteins and decreased E‐cadherin protein expression. Conversely, miR‐671‐5p was found to attenuate the expression of STEAP2 and the activation of the PI3K/Akt/mTOR pathway, as well as the progression of EMT, induced by circHPCAL1 overexpression (Figure 3V). We also found that si‐circHPCAL1 alone had a modest effect on glycolytic levels in BxPC‐3 and PANC‐1 cells via an extracellular acidification rate (ECAR) assay, but combined knockdown of circHPCAL1 with gemcitabine significantly reduced glycolytic activity in PaCa cells (*p* < 0.05) (Figure 3W,X). Collectively, our findings suggest that circHPCAL1 modulates gemcitabine sensitivity in PaCa cells by regulating their glycolytic capacity.

**FIGURE 2:**
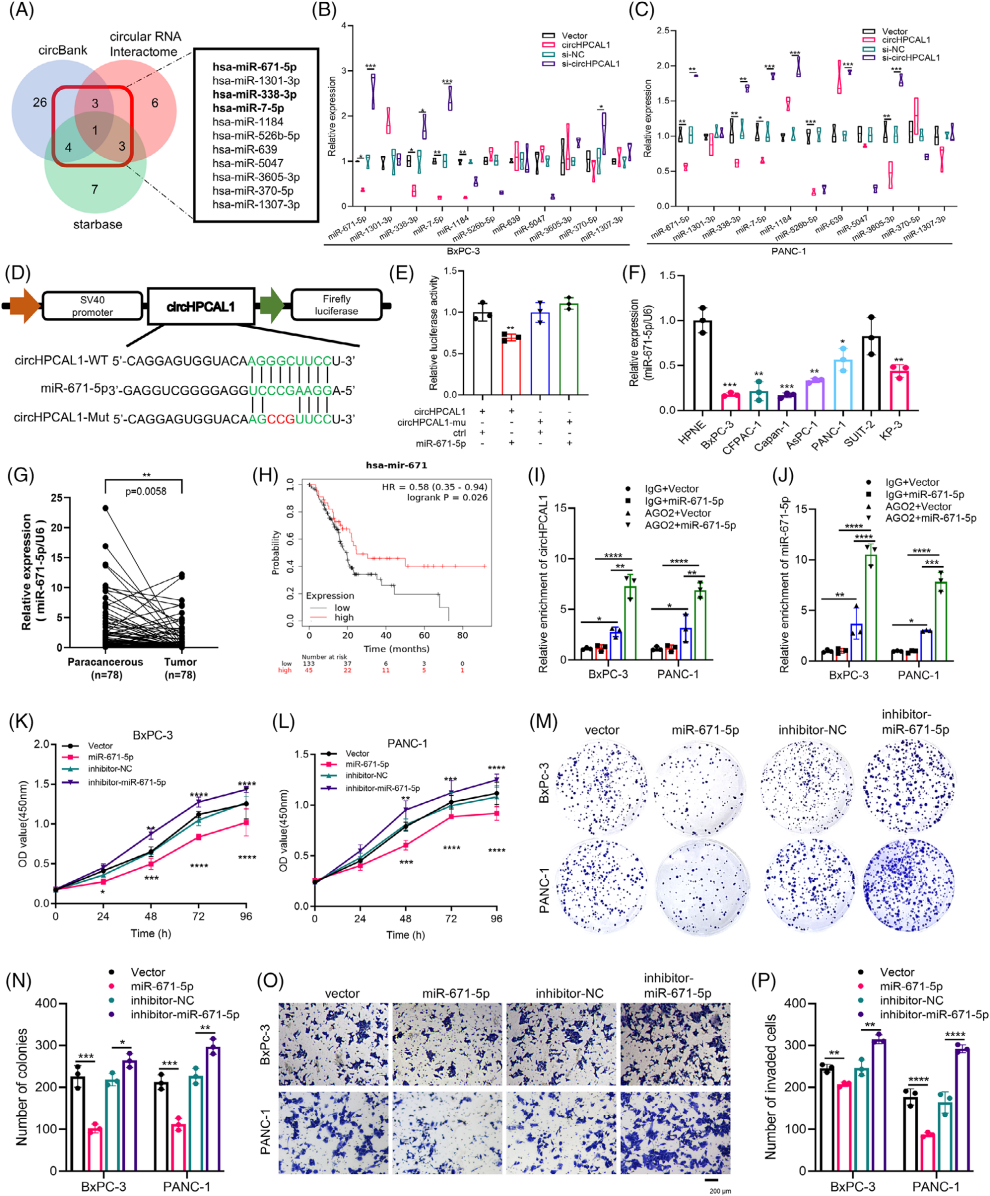
miR‐671‐5p acts as the target of circHPCAL1 and inhibits the proliferation and invasion of pancreatic cancer (PaCa). (A) Target miRNAs of circHPCAL1 were analysed via starBase, circBank, and the circular RNA interactome. (B, C) Quantitative real‐time polymerase chain reaction (qRT‐PCR) verified the expression levels of 11 candidate target miRNAs. (D, E) The dual‐luciferase assay determined the direct binding of miR‐671‐5p and circHPCAL1. (F, G) qRT‒PCR analysis of the expression of potential miRNAs (miR‐671‐5p) of circHPCAL1 in pancreatic tumour tissues and cells. (H) The Kaplan‐Meier analyses evaluated the survival and prognostic value of miR‐671‐5p in PaCa. (I, J) RIP assay to determine the direct interaction between miR‐671‐5p and circHPCAL1. (K, L) The CCK‐8 assays analysed the effects of miR‐671‐5p on the growth of PaCa cells. (M–P) The colony formation (M, N) and Transwell assays (O, P) evaluated the effects of miR‐671‐5p in PaCa cells. * *p* < 0.05, **p < 0.01, ***p < 0.001 and ****p < 0.0001.

**FIGURE 3:**
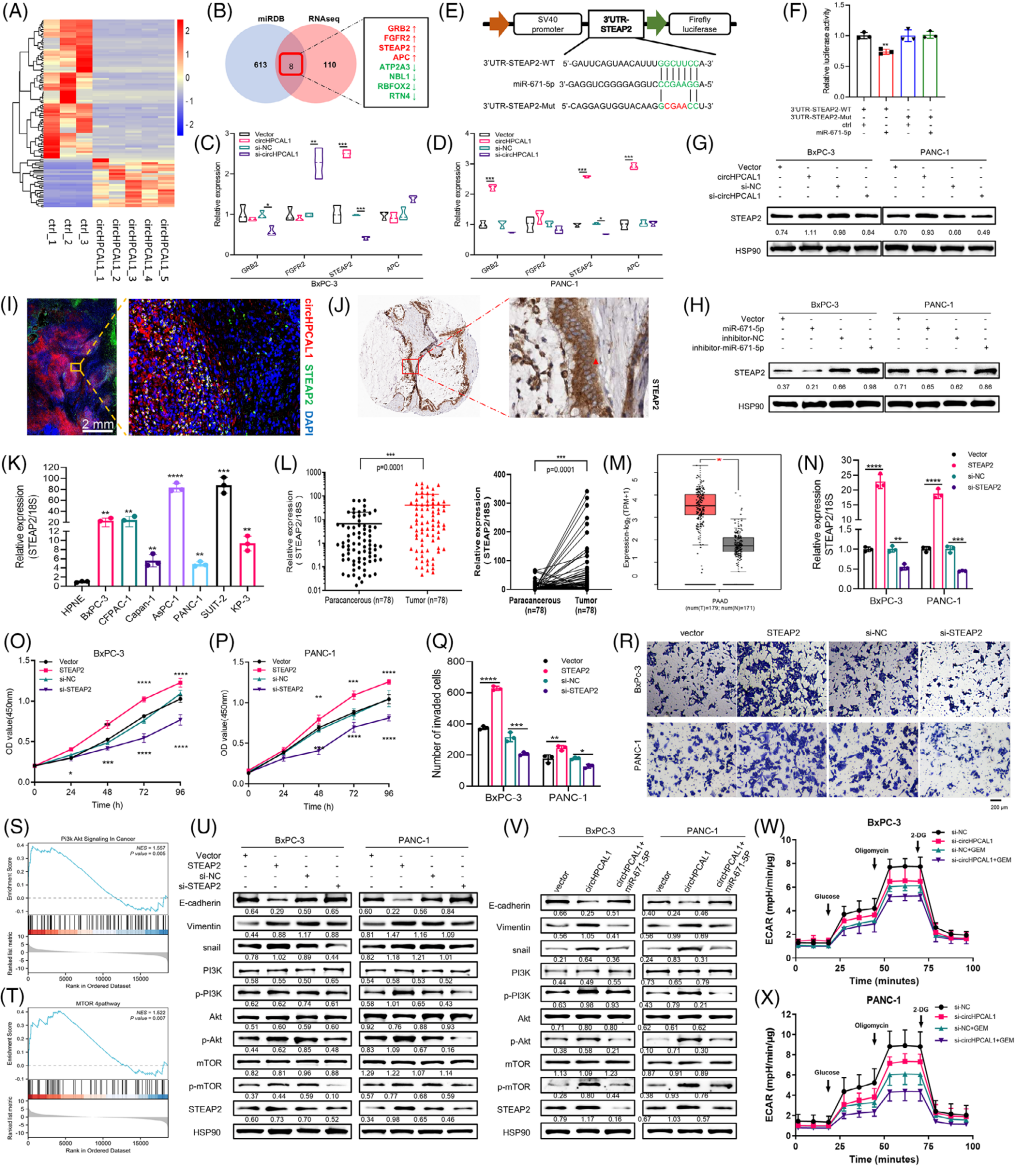
STEAP2 acts as a miR‐671‐5P target and promotes pancreatic cancer (PaCa) cell proliferation and invasion, and circHPCAL1 promotes PaCa progression via the miR‐671‐5p/STEAP2 axis. (A) Heatmap of the RNA‐seq analysis. (B) Venn diagram of the differentially expressed genes according to the RNA‐seq results and the miR‐671‐5p target genes in the miRDB database. (C, D) Quantitative real‐time polymerase chain reaction (qRT‐PCR) verified the expression levels of four potential target mRNAs. (E, F) The dual luciferase assay determined the direct binding of miR‐671‐5p to the 3′UTR of the STEAP2 gene. (G, H) The protein expression level of STEAP2 was analysed in PaCa cells after circHPCAL1 (G) or miR‐671‐5p (H) was overexpressed or knocked down by immunoblots. (I) RNA FISH‐IF assays showed the localisation of circHPCAL1 (red) and STEAP2 protein (green) in PaCa tissues. (J) Representative protein expression levels of STEAP2 in PaCa tissues from the HPA database. (K, L) The expression levels of STEAP2 in pancreatic cells (K) and tumour tissues (L) were analysed by qRT‒PCR. (M) The GEPIA2 website was used to predict the STEAP2 expression. (N) The mRNA expression levels of STEAP2 were analysed in the PaCa cells transfected with the STEAP2 overexpression or siRNA. (O, P) The CCK‐8 assay evaluated the effects of STEAP2 on the growth of PaCa cells. (Q, R) Transwell assays evaluated the effects of miR‐671‐5p on the invasion of PaCa cells. (S, T) Gene functional enrichment analysis of the RNA‐seq data of circHPCAL1‐overexpressing cells. (U, V) The PI3K/Akt/mTOR protein phosphorylation levels and the expression of E‐cadherin, Vimentin, and Snail1 were analysed in the PaCa cells with overexpression and knockdown of STEAP2 (U) or overexpression of circHPCAL1 and miR‐671‐5p in PaCa cells after miR‐671‐5p replenishment (V) by immunoblots. (W, X) Glycolysis assays in PaCa cells after circHPCAL1 knockdown or gemcitabine treatment. * *p* < 0.05, **p < 0.01, ***p < 0.001 and ****p < 0.0001.

Finally, we established an orthotopic xenograft PaCa tumour model using BxPC‐3 cells stably expressing luciferase. The effect of intravenous injection of si‐circHPCAL1 on the PaCa model was then analysed. The luciferase images demonstrated that si‐circHPCAL1 significantly inhibited the growth of PaCa tumours in vivo (Figure 4A,B), although there was no significant difference in body weight between the two groups of mice (Figure 4C). Compared with those in the control group, the expression levels of circHPCAL1, STEAP2, Vimentin, and Snail1 were decreased in the si‐circHPCAL1 group, whereas the expression levels of miR‐671‐5p and E‐cadherin genes were increased (Figure 4D–J). Immunohistochemical (IHC) analyses revealed that the expression of Snail1 in both the cytoplasm and nucleus of tumour cells was markedly reduced in the circHPCAL1‐knockdown group (Figure 4F,K). The expression levels of vimentin (Figure 4G,L) and STEAP2 (Figure 4I,N) in the cytoplasm were also decreased in the circHPCAL1‐knockdown group. In contrast, the expression of E‐cadherin on the cell membrane was markedly greater in the circHPCAL1‐knockdown group than in the control group (Figure 4H,M). Immunoblots further revealed a decrease in the protein expression of STEAP2 in the si‐circHPCAL1 group (Figure 4O). Compared with those in the control group, a significant downregulation of Snail1 and Vimentin, along with an upregulation of E‐cadherin, was observed in the si‐circHPCAL1 group (Figure 4O), which indicates that si‐circHPCAL1 suppresses the EMT process in PaCa by regulation of STEAP2 in vivo. Taken together, our results demonstrate that the activation of EMT in PaCa by circHPCAL1 through the PI3K/AKT/mTOR pathway via the miR‐671‐5p/STEAP2 axis (Figure 4P).

**FIGURE 4:**
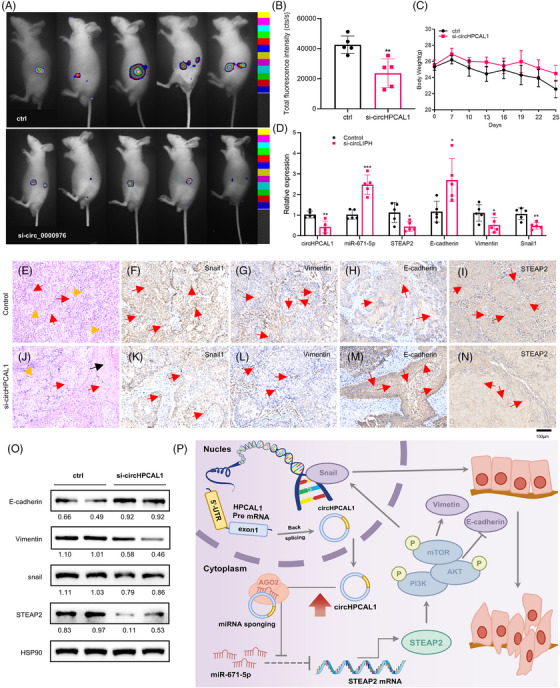
si‐circHPCAL1 represses pancreatic cancer (PaCa) progression in vivo. (A, B) The luciferase images (A) and luciferase intensity assays (B) of PaCa orthotopic models in nude mice treated with si‐circHPCAL1 or controls. (C) Weight monitoring curve of nude mice. (D) Quantitative real‐time polymerase chain reaction (qRT‐PCR) analyses of circHPCAL1, miR‐671‐5p, STEAP2, E‐cadherin, vimentin, and Snail1 mRNA expression in xenograft tumour tissues from mice with or without intravenous injection of si‐circHPCAL1. (E–N) HE staining (E, J) and IHC analyses of SNAI1 (F, K), vimentin (G, L), E‐cadherin (H, M), and STEAP2 (I, N) protein expression in xenograft tumours from control (E–I) and si‐circHPCAL1‐injected (J–N) mice (HE staining: red arrows, tumour cells; green arrows, peritumoral vascular endothelium; black arrows, collagen fibres. IHC: red arrows, positive staining; ×400 magnification). (O) Immunoblots analyses of SNAI1, vimentin, E‐cadherin, and STEAP2 protein expression in xenograft tumour tissues from mice with or without si‐circHPCAL1 injection. (P) Graphical summary of circHPCAL1 activation of the PI3K/Akt/mTOR signalling pathway and the EMT process through the miR‐671‐5p/STEAP2 axis in PaCa. * *p* < 0.05, **p < 0.01, ***p < 0.001 and ****p < 0.0001.

## AUTHOR CONTRIBUTIONS

Jin Wang designed the study. Xiaomeng He, Qiuyue Li, Zehuan Li and Shanshan Liu performed the experiments. Xiaomeng He, Wenchuan Wu and Yang Di collected the clinical samples. Xiaomeng He, Yang Di, Lixiang Sun, Li Xu, Xin Zhang and Mengting Luo analysed the data. Xiaomeng He was responsible for the statistical analysis and drafted the manuscript. Jin Wang and Christopher Corpe revised the manuscript. Jianqing Xu and Xiaoyan Zhang supported funding and resources. All the authors have read and approved the final manuscript.

## CONFLICT OF INTEREST STATEMENT

The authors declare no conflict of interest.

## FUNDING INFORMATION

This research was supported by a grant from the Fujian Provincial Natural Science Foundation of China (2024J011432), China, a grant from the Xiamen Global Talents Fund Project (to Jin Wang), China, a grant from the Fujian Provincial Health and Wellness Science and Technology Program (2024GGB24), and a grant from the Science and Technology Plan Project of Xiamen (3502Z20224012), China.

## ETHICS STATEMENT

All human samples were analysed for the current study with the protocol approved by the Ethics Committees of Zhongshan Hospital (Xiamen) of Fudan University (B2024‐133).

## PATIENT CONSENT STATEMENT

All the authors involved in this manuscript provided consent for publication.

## Supporting information



Supporting information

## Data Availability

The data underlying the findings of this study are available within the article, the online Supporting Information files, and publicly available databases. Further inquiries regarding data requests can be directed to the corresponding authors.
