# Supplementary material for: CircHPCAL1 promotes the progression of pancreatic cancer via the regulation of STEAP2
Source: Clin Transl Med. 2025 Oct 9;15(10):e70501. doi: 10.1002/ctm2.70501 (PMC12508615; doi:10.1002/ctm2.70501)

CircHPCAL1 promotes the progression of pancreatic cancer via the regulation of STEAP2

Xiaomeng He[1, 2], Yang Di[3], Lixiang Sun[1,4], Wenchuan Wu[5], Zehuan Li[5], Qiuyue Li[2], Shanshan Liu[2], Mengting Luo[1], Xin Zhang[1], Li Xu[2], Xiaoyan Zhang[3,6], Jianqing Xu[3,6], Christopher Corpe[7], Jin Wang[1, 4, *]

[1]Central Laboratory, Zhongshan Hospital (Xiamen), Fudan University, Xiamen 361015, China; [2]Shanghai Public Health Clinical Center, Fudan University, 2901 Caolang Road, Jinshan District, Shanghai, China; [3]Department of Pancreatic Surgery, Pancreatic Disease Institute, Huashan Hospital, Shanghai Medical College, Fudan University, Shanghai, China; [4]Xiamen Key Laboratory of Biotherapy, Xiamen 361015, China; [5]Department of General Surgery, Zhongshan Hospital, Fudan University, Shanghai 200032, China; [6]Clinical Research Center for Precision Medicine of Abdominal Tumor of Fujian Province; [7]King's College London, London, Department of Nutritional Science, 150 Stamford Street, Waterloo, London, SE19NH, United Kingdom.

Running Title: CircHPCAL1 promotes the progression of PaCa *via* the regulation of STEAP2

Keywords: Pancreatic cancer, circHPCAL1, miR671-5P, STEAP2

***Correspondence should be addressed to:**

Jin Wang, Ph.D.

Central Laboratory,

Zhongshan Hospital (Xiamen),

Fudan University,

Xiamen 361015, China

Ph: 86-17701869068;

Email: wang.jin@zsxmhospital.com

ORCID ID: orcid.org/0000-0002-0062-2489

Supplemental Information

Materials and methods

Clinical samples

Our study population consisted of patients with pathologically confirmed primary PaCa and normal controls recruited at Zhongshan Hospital (Xiamen) of Fudan University. Normal controls were frequency-matched to cases by age at enrollment, sex, and race. All study subjects provided written informed consent following the protocol approved by the Institutional Review Board of Zhongshan Hospital (Xiamen) of Fudan University (B2024-133). This study analyzed 78 PaCa and paracancerous tissues and plasma samples collected from PaCa patients, as well as 70 normal control plasma samples.

Pancreatic cell lines and cell transfection

Human normal pancreatic ductal epithelial (HPNE) cells were cultured in DMEM supplemented with 5% fetal bovine serum (FBS) (Cat#. 26010074, Gibco), human recombinant epidermal growth factor (EGF), D-glucose, and puromycin. 293T cells and the PaCa cell lines CFPAC-1, Capan-1, SUIT-2, and PANC-1were propagated in DMEM enriched with 10% FBS. BxPC-3, AsPC-1, and KP-3 cells were maintained in RPMI-1640 medium supplemented with 10% FBS. All the cell lines were incubated in a humidified environment at 37°C with 5% CO_2.

The oligonucleotides specifically targeting circHPCAL1 (si-circHPCAL1), STEAP2 (si-STEAP2), and the miR-671-5p inhibitor were designed and synthesized by RiboBio (Guangzhou). The sequence details of the siRNAs are provided in **Table S1**. Moreover, the circHPCAL1 overexpression plasmid, along with its corresponding empty vector

PLC5-ciR, was acquired from Guangzhou Geneseed Biotech Co., Ltd. The overexpression plasmids for STEAP2 (pEGFP-C1) and miR-671-5p (pcDNA3.1) were constructed in our laboratory. The transfection process was carried out *via* the use of Lipofectamine 3000 reagent (Cat$^{\#}$. L3000008, Thermo Fisher), which adhered strictly to the manufacturer's guidelines. Additionally, for functional evaluation, BxPC-3 and PANC-1 cells were subjected to gemcitabine (GEM) (Cat$^{\#}$. G6423, Sigma–Aldrich) treatment.

RNA extraction, purification, and qRT–PCR analysis

Total RNA was isolated from tissues and cells *via* TRIzol reagent (Cat$^{\#}$. 15596026CN; Invitrogen) and an RNA Simple Total RNA Kit (Cat$^{\#}$. DP419; Tiangen Biotech) according to the manufacturers' guidelines. The concentration of total RNA was subsequently evaluated *via* a NanoDrop 2000 spectrophotometer. A PrimeScript RT Reagent Kit (Cat$^{\#}$. RR047A, Takara) and TB Green Premix Ex Taq II (Cat$^{\#}$. RR820A, Takara) were used for quantitative reverse transcription polymerase chain reaction (qRT–PCR) analysis. To standardize the expression levels of genes and circRNAs, 18S rRNA was used as an endogenous control. The miRNA expression levels were normalized to those of U6 as an endogenous control. The relative expression levels of RNA in each sample were calculated via the $2^{-\Delta\Delta Ct}$ method. The primer sequences utilized in this study are shown in **Table S1**.

Functional pathway analysis and target miRNAs for circHPCAL1 analyses.

To gain insight into the functions of differentially expressed circular RNAs (circRNAs), gene ontology (GO) (http://www.geneontology.org) analysis was performed, aiming to provide meaningful annotations for genes and gene products, including biological

processes, cellular components, and molecular functions. Potential target miRNAs for circHPCAL1 were predicted *via* databases including starBase (https://rnasysu.com/encori/)、circBank, circBank (http://www.circbank.cn/), and the circular RNA interactome (https://circinteractome.irp.nia.nih.gov/circular_rna.html). Furthermore, the downstream target genes of circHPCAL1 were predicted *via* the miRDB database (https://www.mirdb.org/) and RNA-seq sequencing results.

Luciferase reporter gene assay

The binding site of miR-671-5p in the 3'-UTR of STEAP2 was cloned and inserted into the pMIR-REPORT luciferase vector. Furthermore, the binding sites of miR-671-5p in circHPCAL1 and the binding sites of miR-671-5p in the 3'-UTR of STEAP2 were targeted for mutation. The sequences of premiR-671-5p, premiR-338-3p, and premiR-7-5p were cloned and inserted into the pcDNA3.1 vector, which was subsequently cotransfected with the luciferase reporter plasmid and the Renilla luciferase reporter vector (pRL-TK) into 293T cells at the designated concentrations (1 μg). After 48 hours of transfection, the luciferase activity was assessed *via* the Promega dual luciferase system (Cat#. E1910, Promega), with Renilla luciferase activity serving as an internal reference.

RNA immunoprecipitation (RIP)

BxPC-3 and PANC-1 cells were lysed in RIPA lysis buffer (Cat#. C500007-0100; Sangon) containing protease and phosphatase inhibitors to prepare the cell supernatant. The supernatant was subsequently incubated with magnetic beads (Cat#. B23202, Selleck) conjugated with an anti-AGO2 antibody (Cat#. 2897, Cell Signaling

Technology) or an anti-human IgG antibody overnight at 4°C. After proteinase K buffer treatment and washing, the immunoprecipitated RNA was extracted with TRIzol reagent and then reverse transcribed. Finally, the levels of circHPCAL1 and miR-671-5p were quantified via qRT-PCR.

Immunoblotting

According to the manufacturer's instructions, total cellular protein was extracted with RIPA lysis buffer and the protein concentration was measured with a BCA protein concentration assay kit. Then, 30 μg of protein lysate was separated on a 10% SDS–PAGE gel. The electrophoretically separated proteins were then transferred onto a nitrocellulose membrane (Cat#. 66485, PALL). The membrane was blocked with TBS-T buffer containing 5% skim milk for an hour, followed by an overnight incubation at 4°C with the primary antibody. The membrane was subsequently incubated with an HRP-conjugated secondary antibody (Sangon) at room temperature for 1.5 hours. Finally, the HRP activity was visualized with an enhanced chemiluminescence (ECL) reagent (Cat#. P0018AFT, Beyotime). The antibodies used in the study included anti-E-cadherin (Cat#. 20874-1-AP, Proteintech), anti-vimentin (Cat#. 60330-1-Ig, Proteintech), anti-Snai1 (Cat#. 13099-1-AP, Proteintech), anti-STEAP2 (Cat#. 20201-1-AP, Proteintech), anti-PI3K (Cat#. 60225-1-Ig, Proteintech), anti-AKT (Cat#. 60203-2-Ig, Proteintech), anti-mTOR (Cat#. 66888-1-Ig, Proteintech), anti-p-PI3K (Cat#. 4228, Cell Signaling Technology), anti-p-AKT (Cat#. 66444-1-Ig, Proteintech), anti-p-mTOR (Cat#. 67778-1-Ig, Proteintech), and anti-Hsp90 (Cat#. 13171-1-AP, Proteintech) antibodies.

Fluorescence in situ hybridization (FISH) and Immunofluorescence (IF) assays

The biotin-labeled probes circHPCAL1 (ACTACACCTGCACGATCTCCAGCAT), 18S (CTGCCTTCCTTGGATGTGGTAGCCGTTTC), and U6 (CACGAATTTGCGTGTCATCCTT) were designed and synthesized by GenePharma (Shanghai). BxPC-3 and PANC-1 cells were plated onto 8-well glass slides (Cat. PEZGS0816, Merck Millipore) and allowed to adhere overnight. Following the manufacturer's protocol, the cells were fixed with 4% paraformaldehyde for 15 minutes, permeabilized with 0.1% Triton X-100, and blocked with 5% bovine serum albumin. The FISH probe was diluted in hybridization solution, denatured at 75°C, and then coupled with SA-Cy3 at 37°C. The prepared FISH probe solution was added to the cells, which were subsequently hybridized overnight at 37°C. After hybridization, the nuclei were stained with DAPI (containing an anti-quenching agent) and mounted. Finally, the samples were observed and analyzed *via* a Leica TCS SP5 (Germany) laser confocal microscope. On another hand, after the human PaCa tissues underwent fixation, embedding, and sectioning into 4 μm slices, the Cy3-labeled circHPCAL1 probe and STEAP2 primary antibody with Alexa Fluor 488-conjugated Goat anti-rabbit IgG secondary antibody for FISH-IF assays were performed to analyze the localization of circHPCAL1 and STEAP2 protein in PaCa tumor tissues by the confocal microscope (Nikon A1, Japan).

CCK8 and colony formation assays

Transfected BxPC-3 and PANC-1 cells were digested with trypsin and resuspended in complete medium. A Cell Counting Kit-8 (CCK8) (Cat$^{\#}$. The E606335–0500; Sangon) method was subsequently used to determine the growth curve. Specifically, the cell suspension was inoculated into a 96-well plate at a density of 5000 cells per well and incubated in a 37°C incubator with 5% CO_2. For five consecutive days, cell viability

was measured by incubating the cells with a CCK8 reaction mixture at 37°C for 2 hours, followed by absorbance measurement at 450 nm to evaluate cell proliferation.

For the plate colony formation assay, the cell suspension was inoculated into 6-well plates at a density of 500 cells per well and maintained in a 37°C, 5% CO_2 incubator for approximately two weeks. After this period, the cells were fixed with 4% paraformaldehyde for 15 minutes and stained with crystal violet for another 15 minutes. The images were then acquired, and the number of clones was determined via Image J (NIH Image) to assess cell population dependence and proliferation.

Cell migration and invasion analyses

Wound healing experiments were conducted to determine the migration ability of the pancreatic cells. Transfected BxPC-3 and PANC-1 cells were cultured in 6-well plates until they reached 90% confluence, streaks were generated on the monolayer by a 200 μL sterile pipette tip, and the cells were then cultured in medium without FBS. Images of the cells were captured at both 0 h and 48 h via an inverted microscope (AMGEVOS, Leica). The migration area was analyzed via ImageJ software.

The invasion ability of cancer cells was determined via the Transwell method. Matrigel (Cat$^{\#}$. 356234, BD Biosciences) was diluted with cold serum-free medium at a ratio of 1:48 and applied to the upper chamber membrane. The membrane was then incubated in a 37°C incubator for 1 hour to allow for solidification and polymerization. Next, 2×10^5 cells were resuspended in 200 μL of serum-free medium and seeded into the upper chamber, while 600 μL of medium containing 10% FBS was added to the lower chamber. After 48 hours of standard culture conditions, the cells were fixed with 4% paraformaldehyde and stained with crystal violet for 15 minutes. The number of cells that had traversed the matrix gel and reached the underside of the membrane was

determined via an inverted microscope (Olympus).

Cytotoxicity assays and glycolysis stress tests

Cell viability was assessed *via* a CCK8 assay after 72 hours of GEM treatment to calculate the half maximum inhibitory concentration (IC_{50}), which was used to verify the effect of circHPCAL1 on the therapeutic efficacy of GEM. After transfection and drug treatment, BxPC-3 and PANC-1 cells (2.5×10^4 cells/well) were plated in Seahorse XFe 24-well cell culture microplates (Seahorse Biosciences) and incubated overnight at 37°C in a 5% CO_2 incubator. The response of cells to continuous injections of glucose (10 mM), oligomycin (1 μM), and 2-DG (1 μM), namely, the extracellular acidification rate (ECAR), was measured with an Agilent Seahorse XFe24 instrument [21]. The ECAR was normalized to the total cellular protein content, and the data are presented as the mean ± standard deviation (SD).

Xenotransplantation experiment

Five-week-old male BALB/c nude mice were purchased from Shanghai Jihui Laboratory Animal Breeding Co., Ltd. BxPC-3 cells (5×10^5) stably expressing luciferase were injected into the pancreas of nude mice to establish a model of PaCa in situ tumor formation. One week later, each group of mice (n = 5) received an intravenous injection of 5 nmol of si-NC or si-circHPCAL1 every three days. During this period, the body weights of the mice were closely monitored. Finally, the mice were imaged *via* the imaging platform of an Ultra-Sensitive Whole Sample Imaging System (NightOWLTM, Berthold) and then analyzed with WinLight 32 software (Berthold) [22]. The animal experimental procedures were approved by the Animal Experiment

Research Ethics Committee of Shanghai Public Health Clinical Center.

Immunohistochemical staining

Immunohistochemical (IHC) staining was performed on formalin-fixed, paraffin-embedded mouse tumor tissue samples using the following primary antibodies, Rabbit anti-SNAIL1 (Cat#: 13099-1-AP, Proteintech), Rabbit anti-Vimentin (Cat#: 10336-1-AP, Proteintech), Rabbit anti-E-cadherin (Cat#: 20874-1-AP, Proteintech), and Rabbit anti-STEAP2 (Cat#: 20201-1-AP, Proteintech). And images were captured using a TissueFAXS bright-field microscope.

Statistics

Statistical analysis was performed with SPSS 26.0 software or GraphPad Prism 9.0 software. The significance of differences was assessed by two-tailed Student's t test (two-group comparison) or two-way ANOVA (multiple-group comparison), and the data are presented as the means ± standard deviations (SDs). $p < 0.05$ was considered statistically significant (* $p < 0.05$, ** $p < 0.01$, *** $p < 0.001$, **** $p < 0.0001$).

Supplementary Tables

Supplementary Table S1. The sequences of the primer and siRNA used in this research.

Gene name	Primer sequences (5'-3')
circHPCAL1-F	GCCTTCAGCATGTACGACCT
circHPCAL1-R	CGACTACACCTGCACGATCT
circHPCAL1-covergent-F	TACGCCAACTTCTTCCCCTA
circHPCAL1-covergent-R	GTACATGCTGAAGGCCCACT
circHPCAL1-divergent-F	GCCTTCAGCATGTACGACCT
circHPCAL1-divergent-R	GCAGCTTGCTGTTCTGTTTG
18S-F	GTAACCCGTTGAACCCCATT
18S-R	CCATCCAATCGGTAGTAGCG
HPCAL1-F	CATGTACGACCTGGACGGC
HPCAL1-R	CTCTTGGCACCTCTGATGAAT
GRB2-F	ATTCCTGCGGGACATAGAACA
GRB2-R	GGTGACATAATTGCGGGGAAAC
FGFR2-F	GGTGGCTGAAAAACGGGAAG
FGFR2-R	AGATGGGACCACACTTTCCATA
STEAP2-F	GTTGTCTCAGCTTGGGCACT
STEAP2-R	CAGAGAGTAAAGAGTCGTAGGGG
APC-F	AAGCATGAAACCGGCTCACAT
APC-R	ATTCGTGTAGTTGAACCCTGA
E-cadherin-F	CGAGAGCTACACGTTCACGG
E-cadherin-R	GGGTGTCGAGGGAAAAATAGG
Vimentin-F	AGTCCACTGAGTACCGGAGAC
Vimentin-R	CATTTCACGCATCTGGCGTTC
Snail1-F	TCGGAAGCCTAACTACAGCGA
Snail1-R	AGATGAGCATTGGCAGCGAG
hsa_miR-671-5p-F	GCGCAGGAAGCCCTGGAGGGG
hsa_miR-671-5p-RT	GTCGTATCCAGTGCAGGGTCCGAGGTATTCGCACTGGATACGACCTCCAG
hsa_miR-1301-3p-F	GCGCTTGCAGCTGCCTGGGAGT
hsa_miR-1301-3p-RT	GTCGTATCCAGTGCAGGGTCCGAGGTATTCGCACTGGATACGACGAAGTC
hsa_miR-338-3p-F	CGCGTCCAGCATCAGTGATT
hsa_miR-338-3p-RT	GTCGTATCCAGTGCAGGGTCCGAGGTATTCGCACTGGATACGACCAACAA
hsa_miR-7-5p-F	CGCGTGGAAGACTAGTGATTTT
hsa_miR-7-5p-RT	GTCGTATCCAGTGCAGGGTCCGAGGTATTCGCACTGGATACGACAACAAC
hsa_miR-1184-F	GCGCCCTGCAGCGACTTGATG
hsa_miR-1184-RT	GTCGTATCCAGTGCAGGGTCCGAGGTATTCGCACTGGATACGACGGAAGC
hsa_miR-526b-5p-F	GCGCCTCTTGAGGGAAGCACT
hsa_miR-526b-5p-RT	GTCGTATCCAGTGCAGGGTCCGAGGTATTCGCACTGGATACGACACAGAA
hsa_miR-639-F	GCGCATCGCTGCGGTTGCGAG
hsa_miR-639-RT	GTCGTATCCAGTGCAGGGTCCGAGGTATTCGCACTGGATACGACACAGCG
hsa_miR-5047-F	GCGCTTGCAGCTGCGGTTG
hsa_miR-5047-RT	GTCGTATCCAGTGCAGGGTCCGAGGTATTCGCACTGGATACGACACCTTA
hsa_miR-3605-3p-F	GCGCCCTCCGTGTTACCTGTC
hsa_miR-3605-3p-RT	GTCGTATCCAGTGCAGGGTCCGAGGTATTCGCACTGGATACGACCTAGAG
hsa_miR-370-5p-F	GCGCCAGGTCACGTCTCTGC
hsa_miR-370-5p-RT	GTCGTATCCAGTGCAGGGTCCGAGGTATTCGCACTGGATACGACGTAACT
hsa_miR-1307-3p-F	GCGCACTCGGCGTGGCGTCG
hsa_miR-1307-3p-RT	GTCGTATCCAGTGCAGGGTCCGAGGTATTCGCACTGGATACGACCACGAC
U6-F	TTCCTCCGCAAGGATGACACGC
U6-RT	GTTGGCTCTGGTGCAGGGTCCGAGGTATTCGCACCAGAGCCAACAAAAATAT
Universal-Reverse	AGTGCAGGGTCCGAGGTATT

F, forwards primer; R, reverse primer.

Supplementary Table S2. Differentially expressed genes in BxPC-3 cell lines with circ_0000976 over-expression

Gene ID	Gene Name	Description	log_2FC	*p* value
ENSG00000134982	APC	APC regulator of WNT signaling pathway	11.25	0.007
ENSG00000177885	GRB2	growth factor receptor bound protein 2	9.88	0.012
ENSG00000158169	FANCC	FA complementation group C	9.22	0.001
ENSG00000136021	SCYL2	SCY1 like pseudokinase 2	8.96	0.048
ENSG00000166897	ELFN2	extracellular leucine rich repeat and fibronectin type III domain containing 2	8.41	0.011
ENSG00000066468	FGFR2	fibroblast growth factor receptor 2	8.00	0.013
ENSG00000171130	ATP6V0E2	ATPase H+ transporting V0 subunit e2	7.84	0.001
ENSG00000039068	CDH1	cadherin 1	7.82	0.026
ENSG00000126653	NSRP1	nuclear speckle splicing regulatory protein 1	7.74	0.005
ENSG00000167615	LENG8	leukocyte receptor cluster member 8	7.30	0.005
ENSG00000167244	IGF2	insulin like growth factor 2	6.99	0.001
ENSG00000172731	LRRC20	leucine rich repeat containing 20	5.55	0.001
ENSG00000161813	LARP4	La ribonucleoprotein 4	5.08	0.001
ENSG00000157214	STEAP2	STEAP2 metalloreductase	5.02	0.001
ENSG00000115756	HPCAL1	hippocalcin like 1	4.94	0.001
ENSG00000102471	NDFIP2	Nedd4 family interacting protein 2	4.84	0.002
ENSG00000163468	CCT3	chaperonin containing TCP1 subunit 3	4.48	0.020
ENSG00000111358	GTF2H3	general transcription factor IIH subunit 3	4.31	0.040
ENSG00000197746	PSAP	prosaposin	4.31	0.041
ENSG00000125743	SNRPD2	small nuclear ribonucleoprotein D2 polypeptide	4.18	0.027
ENSG00000164347	GFM2	GTP dependent ribosome recycling factor mitochondrial 2	4.15	0.013
ENSG00000171604	CXXC5	CXXC finger protein 5	4.04	0.010
ENSG00000197969	VPS13A	vacuolar protein sorting 13 homolog A	4.03	0.009
ENSG00000125971	DYNLRB1	dynein light chain roadblock-type 1	3.87	0.025
ENSG00000067596	DHX8	DEAH-box helicase 8	3.41	0.002
ENSG00000140374	ETFA	electron transfer flavoprotein subunit alpha	3.34	0.004
ENSG00000006432	MAP3K9	mitogen-activated protein kinase kinase kinase 9	2.92	0.003
ENSG00000158747	NBL1	NBL1, DAN family BMP antagonist	-2.70	0.034
ENSG00000121067	SPOP	speckle type BTB/POZ protein	-2.79	0.043
ENSG00000080345	RIF1	replication timing regulatory factor 1	-2.94	0.004
ENSG00000171490	RSL1D1	ribosomal L1 domain containing 1	-3.04	0.030
ENSG00000167904	TMEM68	transmembrane protein 68	-3.04	0.041
ENSG00000126749	EMG1	EMG1 N1-specific pseudouridine methyltransferase	-3.07	0.023
ENSG00000100320	RBFOX2	RNA binding fox-1 homolog 2	-3.09	0.015
ENSG00000171222	SCAND1	SCAN domain containing 1	-3.13	0.022
ENSG00000137760	ALKBH8	alkB homolog 8, tRNA methyltransferase	-3.29	0.014
ENSG00000144161	ZC3H8	zinc finger CCCH-type containing 8	-3.31	0.009
ENSG00000111711	GOLT1B	golgi transport 1B	-3.33	0.040
ENSG00000158882	TOMM40L	translocase of outer mitochondrial membrane 40 like	-3.34	0.021
ENSG00000164904	ALDH7A1	aldehyde dehydrogenase 7 family member A1	-3.39	0.000
ENSG00000107829	FBXW4	F-box and WD repeat domain containing 4	-3.55	0.025
ENSG00000100814	CCNB1IP1	cyclin B1 interacting protein 1	-3.58	0.022
ENSG00000089053	ANAPC5	anaphase promoting complex subunit 5	-3.74	0.010
ENSG00000137547	MRPL15	mitochondrial ribosomal protein L15	-3.82	0.012
ENSG00000126777	KTN1	kinectin 1	-3.86	0.011
ENSG00000143393	PI4KB	phosphatidylinositol 4-kinase beta	-3.95	0.028
ENSG00000105223	PLD3	phospholipase D family member 3	-4.02	0.041
ENSG00000119335	SET	SET nuclear proto-oncogene	-4.06	0.015
ENSG00000177853	ZNF518A	zinc finger protein 518A	-4.14	0.015
ENSG00000143570	SLC39A1	solute carrier family 39 member 1	-4.20	0.042
ENSG00000241973	PI4KA	phosphatidylinositol 4-kinase alpha	-4.21	0.048
ENSG00000136854	STXBP1	syntaxin binding protein 1	-4.29	0.048

ENSG00000145555	MYO10	myosin X	-4.31	0.012
ENSG00000135390	ATP5MC2	ATP synthase membrane subunit c locus 2	-4.31	0.017
ENSG00000134186	PRPF38B	pre-mRNA processing factor 38B	-4.46	0.029
ENSG00000108439	PNPO	pyridoxamine 5'-phosphate oxidase	-4.52	0.010
ENSG00000197879	MYO1C	myosin IC	-4.63	0.039
ENSG00000168734	PKIG	cAMP-dependent protein kinase inhibitor gamma	-4.64	0.001
ENSG00000119787	ATL2	atlastin GTPase 2	-4.66	0.042
ENSG00000178927	CYBC1	cytochrome b-245 chaperone 1	-4.86	0.003
ENSG00000119403	PHF19	PHD finger protein 19	-4.94	0.010
ENSG00000147130	ZMYM3	zinc finger MYM-type containing 3	-4.96	0.009
ENSG00000003436	TFPI	tissue factor pathway inhibitor	-4.96	0.024
ENSG00000168916	ZNF608	zinc finger protein 608	-5.07	0.019
ENSG00000165675	ENOX2	ecto-NOX disulfide-thiol exchanger 2	-5.10	0.002
ENSG00000068724	TTC7A	tetratricopeptide repeat domain 7A	-5.16	0.020
ENSG00000155755	TMEM237	transmembrane protein 237	-5.22	0.003
ENSG00000127125	PPCS	phosphopantothenoylcysteine synthetase	-5.30	0.001
ENSG00000284194	SCO2	synthesis of cytochrome C oxidase 2	-5.35	0.004
ENSG00000163754	GYG1	glycogenin 1	-5.46	0.015
ENSG00000154217	PITPNC1	phosphatidylinositol transfer protein cytoplasmic 1	-5.50	0.008
ENSG00000125863	MKKS	McKusick-Kaufman syndrome	-5.62	0.006
ENSG00000143545	RAB13	RAB13, member RAS oncogene family	-5.71	0.015
ENSG00000104419	NDRG1	N-myc downstream regulated 1	-5.72	0.012
ENSG00000135317	SNX14	sorting nexin 14	-5.77	0.000
ENSG00000065154	OAT	ornithine aminotransferase	-5.78	0.009
ENSG00000129422	MTUS1	microtubule associated scaffold protein 1	-5.90	0.013
ENSG00000142784	WDTC1	WD and tetratricopeptide repeats 1	-5.91	0.001
ENSG00000198646	NCOA6	nuclear receptor coactivator 6	-5.92	0.016
ENSG00000076108	BAZ2A	bromodomain adjacent to zinc finger domain 2A	-6.02	0.001
ENSG00000170525	PFKFB3	6-phosphofructo-2-kinase/fructose-2,6-biphosphatase 3	-6.03	0.020
ENSG00000161203	AP2M1	adaptor related protein complex 2 subunit mu 1	-6.05	0.017
ENSG00000197535	MYO5A	myosin VA	-6.09	0.001
ENSG00000074370	ATP2A3	ATPase sarcoplasmic/endoplasmic reticulum Ca2+ transporting 3	-6.24	0.022
ENSG00000103534	TMC5	transmembrane channel like 5	-6.34	0.005
ENSG00000153187	HNRNPU	heterogeneous nuclear ribonucleoprotein U	-6.36	0.008
ENSG00000122643	NT5C3A	5'-nucleotidase, cytosolic IIIA	-6.41	0.007
ENSG00000140416	TPM1	tropomyosin 1	-6.56	0.010
ENSG00000070540	WIPI1	WD repeat domain, phosphoinositide interacting 1	-6.70	0.041
ENSG00000171204	TMEM126B	transmembrane protein 126B	-6.77	0.027
ENSG00000140718	FTO	FTO alpha-ketoglutarate dependent dioxygenase	-6.79	0.003
ENSG00000115310	RTN4	reticulon 4	-6.84	0.014
ENSG00000173039	RELA	RELA proto-oncogene, NF-kB subunit	-6.90	0.001
ENSG00000198121	LPAR1	lysophosphatidic acid receptor 1	-7.05	0.012
ENSG00000117139	KDM5B	lysine demethylase 5B	-7.09	0.015
ENSG00000149782	PLCB3	phospholipase C beta 3	-7.34	0.002
ENSG00000170365	SMAD1	SMAD family member 1	-7.45	0.046
ENSG00000179115	FARSA	phenylalanyl-tRNA synthetase subunit alpha	-7.49	0.003
ENSG00000158856	DMTN	dematin actin binding protein	-7.55	0.006
ENSG00000085224	ATRX	ATRX chromatin remodeler	-7.70	0.003
ENSG00000101972	STAG2	stromal antigen 2	-8.21	0.000
ENSG00000006468	ETV1	ETS variant transcription factor 1	-8.34	0.022
ENSG00000153048	CARHSP1	calcium regulated heat stable protein 1	-8.63	0.012
ENSG00000058272	PPP1R12A	protein phosphatase 1 regulatory subunit 12A	-8.76	0.001
ENSG00000087460	GNAS	GNAS complex locus	-8.94	0.016
ENSG00000120756	PLS1	plastin 1	-9.03	0.015
ENSG00000039560	RAI14	retinoic acid induced 14	-9.17	0.001
ENSG00000188177	ZC3H6	zinc finger CCCH-type containing 6	-9.17	0.000
ENSG00000179222	MAGED1	MAGE family member D1	-9.22	0.014

ENSG00000119139	TJP2	tight junction protein 2	-9.57	0.002
ENSG00000118263	KLF7	Kruppel like factor 7	-9.64	0.001
ENSG00000137509	PRCP	prolylcarboxypeptidase	-9.73	0.000
ENSG00000117632	STMN1	stathmin 1	-10.06	0.008
ENSG00000101871	MID1	midline 1	-10.39	0.000
ENSG00000106290	TAF6	TATA-box binding protein associated factor 6	-10.49	0.002
ENSG00000241685	ARPC1A	actin related protein 2/3 complex subunit 1A	-11.05	0.001
ENSG00000178209	PLEC	plectin	-12.80	0.000
ENSG00000124422	USP22	ubiquitin specific peptidase 22	-13.18	0.01

Note. FC: fold change

Supplementary Figures

Supplemental Figure S1. Expression levels of circRNAs derived from plasma exosomes in PaCa patient 'tissues and function of circHPCAL1. **(A-C)** The migration ability of PaCa cells was evaluated by wound healing (bar = 200 μm) assays. * $P < 0.05$, ** $P < 0.01$, *** $P < 0.001$, **** $P < 0.0001$.

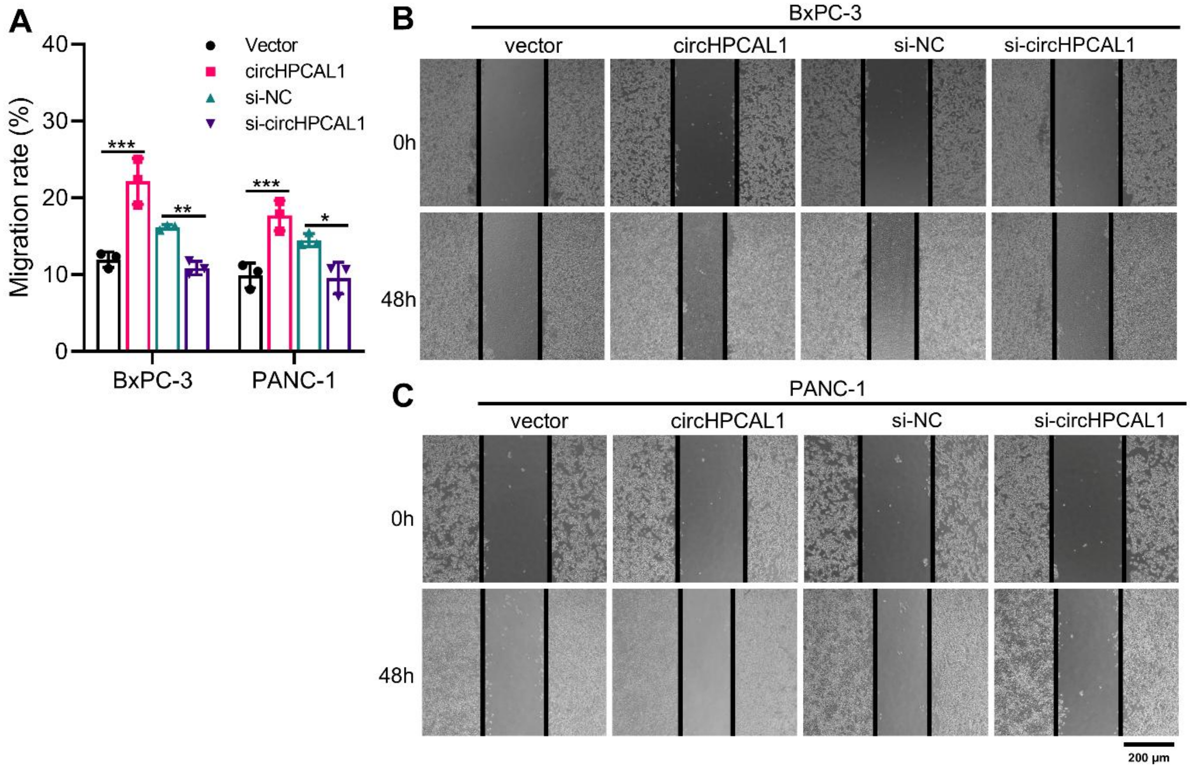

Supplemental Figure S2. miR-671-5P acts as a target of circHPCAL1 and inhibits PaCa growth and migration. **(A, B)** dual luciferase assay was used to determine the direct binding of potential miRNAs (miR-671-5p, miR-338-3p, and miR-7-5p) to circHPCAL1. **(C, D)** qRT-PCR analysis of the expression of miR-338-3p and miR-7-5p in PaCa cells and pancreatic cells. **(E-G)** Wound healing assay was used to evaluate the effects of miR-671-5p overexpression and knockdown on the migration ability of PaCa cells. * P < 0.05, **P < 0.01, ***P < 0.001, ****P < 0.0001.

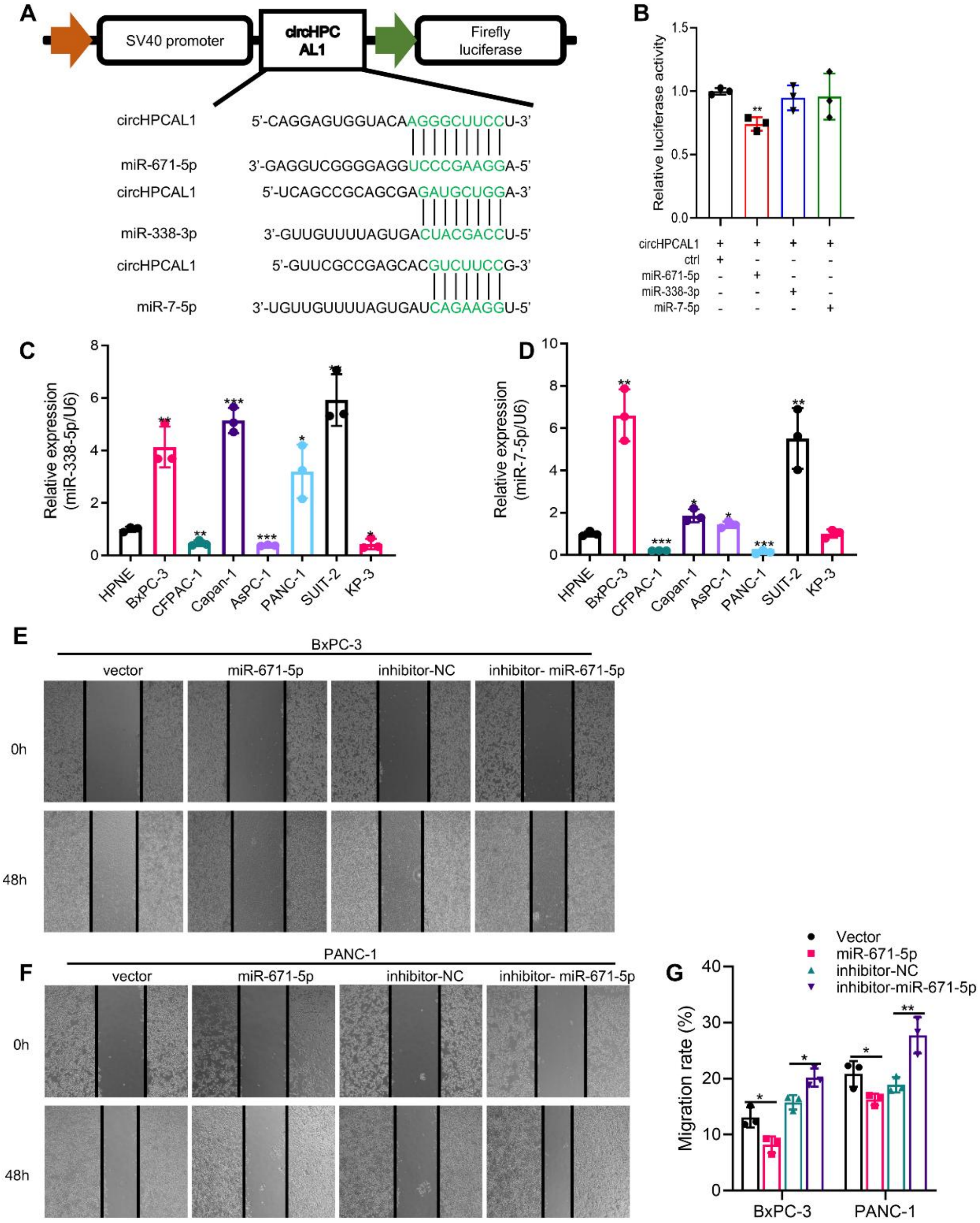

Supplemental Figure S3. STEAP2 promotes PaCa cell growth and migration.

(A, B) The colony formation assay was used to evaluate the effects of miR-671-5p overexpression and knockdown on the proliferation of PaCa cells. **(C-E)** The wound healing assay was used to evaluate the effects of STEAP2 overexpression and knockdown on the migration abilities of PaCa cells. * P < 0.05, **P < 0.01, ***P < 0.001, ****P < 0.0001.

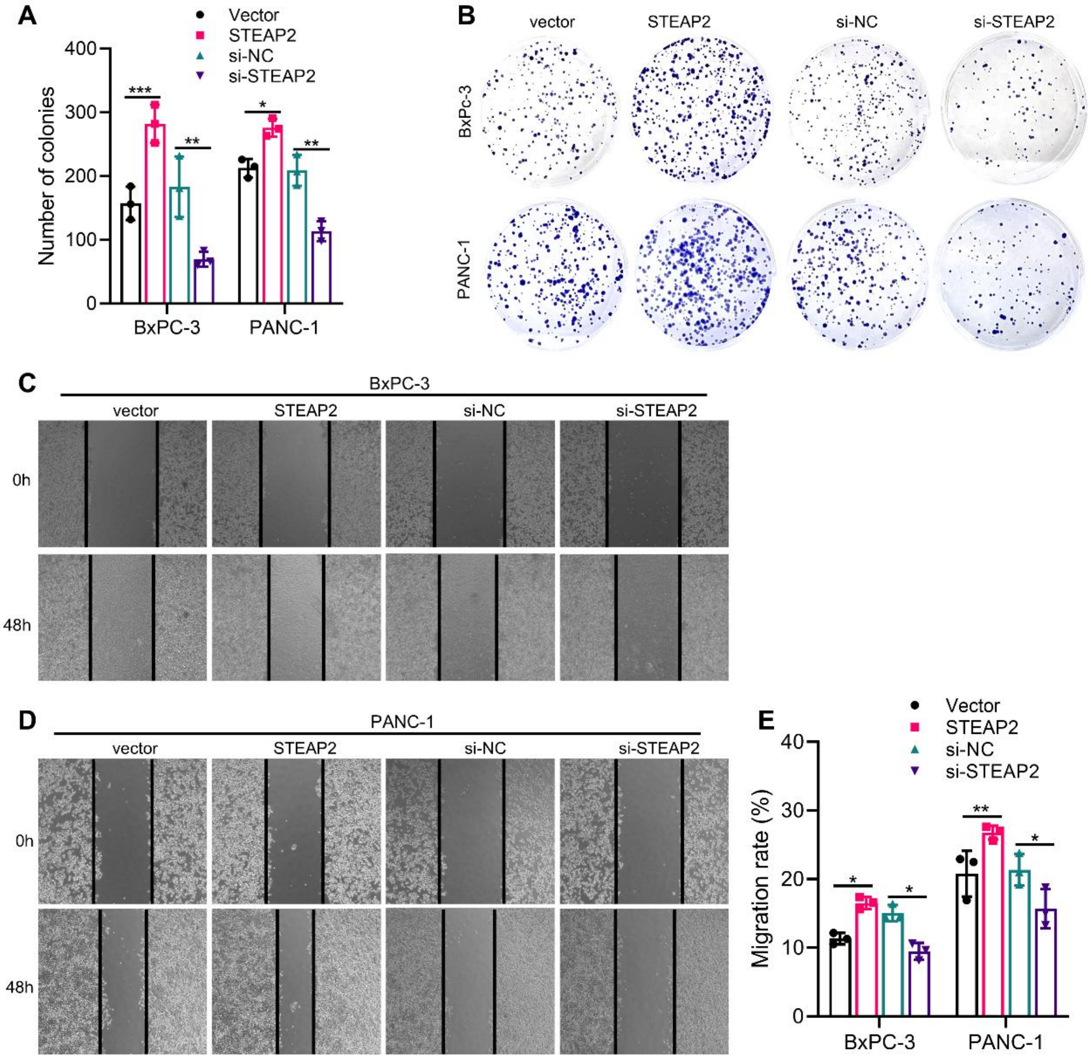

Supplement: Supplementary file 1 — Supporting information [file CTM2-15-e70501-s001.pdf]
